# Synthesis and Study of Gas Transport Properties of Polymers Based on Macroinitiators and 2,4-Toluene Diisocyanate

**DOI:** 10.3390/membranes9030042

**Published:** 2019-03-20

**Authors:** Ilsiya M. Davletbaeva, Ilnaz I. Zaripov, Alexander I. Mazilnikov, Ruslan S. Davletbaev, Raphael R. Sharifullin, Artem A. Atlaskin, Tatyana S. Sazanova, Ilya V. Vorotyntsev

**Affiliations:** 1 Department of Synthetic rubber, Kazan National Research Technological University, 68 K. Marx str., 420015 Kazan, Russia; davletbaeva09@mail.ru (I.M.D.); mazillove001@gmail.com (A.I.M.); 2 Department for Materials Science, Welding and Industrial Safety, Kazan National Research Technical University, n.a. A.N. Tupolev, 10 K. Marx str., 420111 Kazan, Russia; darus@rambler.ru; 3 Laboratory of Scientific and Research Center, PJSC Nizhnekamskneftekhim, 23 Sobolekovskaya str., 423574 Nizhnekamsk, Russia; rafael_sharif@mail.ru; 4 Laboratory of Membrane and Catalytic Processes, Nizhny Novgorod State Technical University n.a. R.E. Alekseev, 24 Minin str., 603950 Nizhny Novgorod, Russia; atlaskin@gmail.com (A.A.A.); yarymova.tatyana@yandex.ru (T.S.S.); ilyavorotyntsev@gmail.com (I.V.V.)

**Keywords:** macroinitiator, 2,4-toluene diisocyanate, microporous block copolymers, gas transport, membrane, gas separation

## Abstract

Nowadays, block copolymers hold great promise for the design of novel membranes to be applied for the membrane gas separation. In this regard, microporous block copolymers based on a macroinitiator with an anionic nature, such as potassium-substituted block copolymers of propylene oxide and ethylene oxide (PPEG) and 2,4-toluene diisocyanate (TDI), were obtained and investigated as effective gas separation membranes. The key element of the macromolecular structure that determines the supramolecular organization of the studied polymers is the coplanar blocks of polyisocyanates with an acetal nature (O-polyisocyanate). In the present research, the influence of the content of peripheral polyoxyethylene (POE) blocks in PPEG on the supramolecular structure processes and gas transport characteristics of the obtained polymers based on PPEG and TDI was investigated. According to the study of polymers if the POE block content is 15 wt %, the polyoxypropylene segments are located in the internal cavity of voids formed by O-polyisocyanate blocks. When the POE block content is 30 wt %, the flexible chain component forms its own microphase outside the segregation zone of the rigid O-polyisocyanate blocks. The permeability for polar molecules, such as ammonia or hydrogen sulfide, significantly exceeds the permeability values obtained for non-polar molecules He, N_2_ and CH_4_. A relatively high permeability is also observed for carbon dioxide. At the same time, the content of POE blocks has a small effect on the permeability for all studied gases. The diffusion coefficient increases with an increase in the POE block content in PPEG for all studied gases.

## 1. Introduction

Due to the constantly growing requirements for the characteristics of substances, such as quality (purity) and environmental friendliness, the design and development of energy-efficient methods and purification technologies have become increasingly popular [1,2,3,4]. In the field of chemical engineering of gas purification, the membrane-based separation technique may be considered as an attractive alternative to conventional energy-intensive processes, such as sorption, crystallization, distillation etc. The ideal membrane process would allow for high selective separation due to the physicochemical features of the material [5,6,7], which passes one component of the gas mixture and rejects others in a continuous steady-state regime. Moreover, the membrane-based process provides separation without any phase transition, does not require heat supply or removal at ambient temperatures. Furthermore, apparatuses should be characterized by simple hardware design. Thus, the high purification of gases is a potential separation field in which membrane technology can be successfully applied individually and as part of hybrid processes [8,9,10,11].

Block copolymers can potentially be used to obtain new membrane materials that can be applied for the selective separation of gases. Poly(styrene-butadiene-styrene) block copolymers were the most studied in this series [12,13,14,15]. According to these works, the degree of the microphase separation of rigid and flexible blocks and the particularity of the supramolecular organization of macrochains significantly affect the gas transport characteristics.

At the same time, the gas selectivity can be improved by adding a third segment to diblock copolymers [16,17]. The properties of the triblock copolymer, poly(styrene-ethylene oxide-styrene) and its mixtures with polyethylene glycol as a membrane for the release of CO_2_ were studied in [18,19]. The selectivity of such membranes to CO_2_ is due to the unusually high affinity of CO_2_ for PEG. By changing the morphology of triblock copolymers containing PEG and the affinity between the copolymer, the selectivity to CO_2_/gas vapors was significantly improved. In reference [20], the comparison of existing membranes based on the block and random polymers of PEG was performed. It turned out that when the PEG content increases, the permeability of membranes decreases but selectivity increases. At the same time, the membranes based on random polymers showed the best selectivity with a PEG content of 20% (CO_2_/CH_4_ = 37.31 and H_2_/CH_4_ = 33.44).

The interesting results related to the relationship between the structure and gas transport characteristics of polyurethane (PU) films obtained on the basis of polyethers and polyesters are presented in references [21,22,23,24,25,26,27,28]. Gas permeability through polyurethane-based membranes can be controlled by changing the ratio of rigid and flexible segments [29,30]. When the content of rigid segments increases, the gas permeability of PU decreases due to a decrease in free volume, segmental mobility and consequently, diffusion [31,32,33,34,35,36,37]. On the contrary, an increase in the content of flexible segments in PU leads to the efficiency of microphase separation and an increase in their gas permeability [38,39]. Moreover, it was revealed that the nature of flexible segments was responsible for the efficiency of gas transportation through membranes [40,41,42,43,44,45]. There is high selectivity for polyurethane membranes obtained from polyethylene glycol but there is low gas permeability for CO_2_. Thus, when the permeability for carbon dioxide is 150 Barrer, the selectivity of CO_2_/N_2_ mixture can reach 50, which is comparable to the separation efficiency of commercial Polyactive [43,44] and Pebax 1657 membranes [20,44]. Nonetheless, the practical use of polyurethane-based membranes is limited by weak thermomechanical stability and their low resistance to plasticization during gas separation. It was found that the branching created in their topological macromolecular structure can have a significant impact on the gas transport properties of polyurethanes. In such a way, the polyurethanes derived from hyperbranched amino ethers of boric acid can show high permeability in combination with high selectivity during the separation of gas mixtures containing ammonia [6,8].

Thus, a change in the nature and ratio of flexible and rigid blocks leads to a change in the properties of the polymer, including those related to gas transport. The use of PEG as a hydrophilic flexible chain component is an important element of the impact on the gas transport characteristics of membrane materials based on block copolymers.

Apart from the gas transport properties of polymeric membranes for the separation of gas mixtures containing methane, nitrogen, carbon dioxide, hydrogen and helium, which have been widely investigated, the processes of the purification of polar and aggressive gases, including ammonia and hydrogen, has not been broadly presented in the literature.

**Figure 1:**
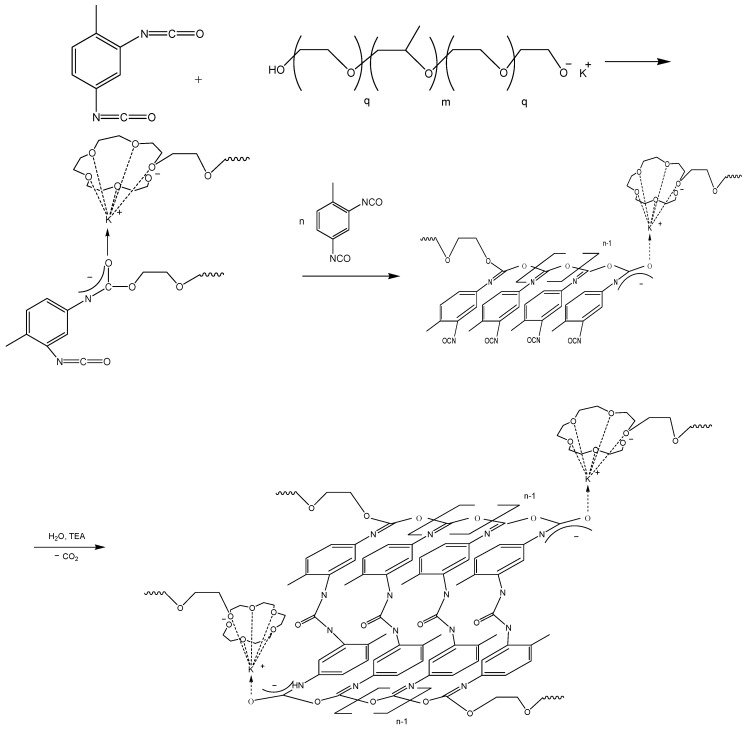
Scheme of O-polyisocyanate blocks formation.

Previous works [45,46,47,48] have described microporous polymers and determined that the specific characteristics of the supramolecular structure of the former are due to the polyisocyanate blocks of acetal nature (O-polyisocyanates) as a rigid chain component. Figure 1 shows the scheme for obtaining such polymers. This scheme is based on the polyaddition reaction of 2,4-toluene diisocyanate (TDI) activated by the macroinitiator (MI), which is basically a potassium-substituted block copolymer of propylene and ethylene oxide (PPEG).

The flexible chain component in these polymers is a block copolymer of propylene and ethylene oxides. Considering that polyoxyethylene (POE) and polyoxypropylene blocks play an important role in gas separation processes, the purpose of this study is to research the influence of the peripheral POE block content in PPEG on supramolecular structure processes, morphology, chemical structure of the inner surface of micropores and gas transport characteristics of polymers obtained on the basis of PPEG and TDI for gases, including polar and aggressive gases (ammonia and hydrogen sulfide).

## 2. Materials and Methods

### 2.1. Materials

The block copolymer of propylene oxide with ethylene oxide (PPEG) with the formula of HO[CH_2_CH_2_O]_n_[CH_2_(CH_3_)CH_2_O]_m_[CH_2_CH_2_O]_n_K and molecular weight of 4200 g/mol, containing 15, 20, 30 and 40 wt % of peripheral POE blocks, were synthesized in the laboratory of Scientific and Research Center, PJSC Nizhnekamskneftekhim (Nizhnekamsk, Russia), which also had a content of potassium alcholate groups that was 10.9% of the total number of functional groups. The 2,4-toluene diisocyanate 98% (TDI) was purchased from Sigma-Aldrich (St. Louis, MO, USA). PPEG was additionally dried at a reduced pressure and at an elevated temperature of 353 K to reduce the moisture concentration to 0.01%.

In the gas separation investigation of the prepared polymer gases (methane, hydrogen sulfide, carbon dioxide, helium and nitrogen), all gases used had a purity of no less than 99.995% (NII KM, Nizhny Novgorod, Russia). High purity ammonia (99.99999%; Firm HORST Ltd., Moscow, Russia) was employed for permeability studies.

### 2.2. Synthesis of Polymers Based on TDI and PPEG

The reaction was carried out in toluene at 20 °C in a flask equipped with a reflux condenser. The polymerization process proceeded with the constant stirring using a magnetic stirrer. The flask contained PPEG (1 g), toluene (7.9 g), bisphenol A (0.004 g) and acetic acid (0.007 g). The reaction mass was mixed at a given temperature until the complete dissolution of PPEG took place. After this, TDI (0.62 g) at a molar ratio of [PPEG]:[TDI] = 1:15 was introduced. Five minutes after being mixing with TDI, the reaction system was loaded with triethylamine (0.0072 g) and water (0.001 g). Ten minutes after the reaction mass was dispensed to Petri dish, it was cured at room temperature for 72 h.

### 2.3. Polymer Characterization

#### 2.3.1. Tensile Stress–Strain Measurements

Tensile stress–strain measurements were obtained from the film samples with dimensions of 40 mm × 15 mm using Universal Testing Machine Inspekt mini (Hegewald & Peschke Meß- und Prüftechnik GmbH, Nossen, Germany) at 293 ± 2 K and 1 kN. The crosshead speed was set at 50 mm/min and the test continued until sample failure. A minimum of five tests was analyzed for each sample and the average values were reported.

#### 2.3.2. Fourier Transform Infrared (FTIR) Spectroscopy Analysis

The FTIR spectra were recorded on an InfraLUM FT 08 Fourier transform spectrometer (Lumex, St. Petersburg, Russia) using the attenuated total reflection technique in the spectral range of 3800–400 cm^−1^. The spectral resolution was 2 cm^−1^ and the number of scans was 10.

#### 2.3.3. Thermomechanical Analysis

The thermomechanical curves of polymer samples were obtained using TMA 402 F (Netzsch, Selb, Germany) thermomechanical analyzer in the compression mode. The thickness of the sample was 2 mm, the rate of heating was 3 K/min from room temperature to 540 K in the static mode and the load was 2 N.

#### 2.3.4. Thermal Gravimetric Analysis

Thermal gravimetric analysis (TGA) was performed using STA-600 TGA–DTA combined thermal analyzer (Perkin Elmer, Waltham, MA, USA). The samples (0.1 g) were loaded in alumina pans and heated from 297 to 870 K at a rate of 5 K/min in a nitrogen atmosphere.

#### 2.3.5. Atomic Force Microscopy Topology Analysis

The atomic force microscopy (AFM) was used to reveal the surface morphology of samples. All AFM measurements were obtained using an atomic force microscope (SPM-9700, Shimadzu, Kyoto, Japan) and silicon vibration cantilevers (POINTPROBE FMR-20, Neuchaatel, Switzerland) with a typical tip curvature radius of no greater than 8 nm. The lateral scan area was of up to 5 μm × 5 μm. The microscopic images were obtained at a resolution of 256 × 256. Membrane samples were attached to the metal sample discs using adhesive carbon tabs (SPI Supplies Division of Structure Probe Inc., West Chester, PA, USA). The AFM images were obtained for three samples of each material. The measurements were all carried out under ambient conditions using the tapping mode of imaging.

#### 2.3.6. Water Adsorption

Water adsorption was determined by the gravimetric method. A sample of a certain size was cut out from the obtained polymer. The resulting sample was weighed on an analytical balance. The measuring cup loaded with distilled water before the sample was placed in water and fixed. After a specified amount of time, the samples were removed, the remaining water from the surface was removed using filter paper and the sample was weighed. Water adsorption (B) is calculated as a percentage using the following formula:

B = (m_s_ − m)/m

where m is the mass of the sample before the test, g; and m_s_ is the mass of the sample after the test. The arithmetic average of the results of the five tests was determined as the main result.

#### 2.3.7. Permeability Measurements

Single gas permeances tests through the obtained membranes (polymeric films) were performed according to the Daynes-Barrer technique in a constant-volume variable-pressure apparatus for gas permeability measurements at the initial transmembrane pressure difference of 110 kPa and at ambient temperature (25 °C).

**Figure 2:**
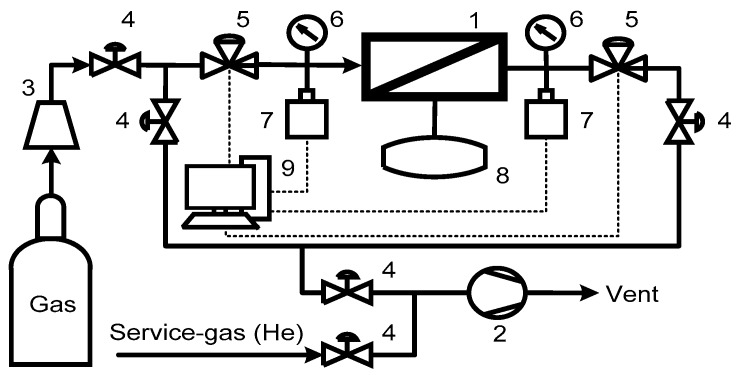
Schematic of experimental setup for single gas permeability test: 1 = permeation cell; 2 = vacuum station; 3 = pressure reducer; 4 = manual valves; 5 = pneumatic valves; 6 = pressure gauges; 7 = pressure transducers; 8 = additional volume for the permeate side; and 9 = workstation.

The experimental setup presented in Figure 2 was designed for high precision single gas time-lag permeation tests. The setup is comprised of a typical permeation cell made of AISI316 stainless steel with PTFE sealing, a vacuum turbomolecular station HiCube 80 Eco (Pfeiffer Vacuum, Asslar, Germany). The initial vacuum generated in the system was 4 × 10^−5^ Pa. The membrane area was 2.54 cm^2^. The feed- and permeate-side pressures were monitored by high precision pressure sensors PT 5000 (A-Flow, Paris, France) at an accuracy of 0.5% of the span (for the feed side) and MKS Baratron 750B 0–100 torr pressure transducer (MKS Instruments, Andover, MA, USA) with an accuracy of 1% of the reading (for the permeate side). The constant feed pressure was maintained at 110 kPa and the permeate pressure evolution was recorded with a sampling rate of 10 ms. Each single gas test was repeated at least three times. A radial membrane permeation cell (position 1 on Figure 2) with a porous mechanical support was used, which was vacuum-degassed before each measurement.

The single gas permeability coefficients for helium, nitrogen, methane, carbon dioxide, hydrogen sulfide and ammonia through each sample were measured at least three times. After the ammonia analysis, the permeability coefficients of helium and nitrogen were measured again to evaluate the chemical resistance of polymer. All experiments were carried out following safety precautions, which are specified in ISO/TC 58/SC 4 and ISO/TC 161. After the investigation of ammonia gas transport properties, the permeability coefficients of helium and nitrogen did not change.

The permeability coefficient P was calculated according to:

(1) $$P=\frac{{\mathrm{Vp}}_{2}T_{0}}{V_{m}P_{0}T}\frac{l}{S\tau \left(p_{1}-p_{2}\right)}$$

where V is the permeate side volume, m^3^; V_m_ is the molar volume, m^3^/mol; $p_{2}$ is the permeate side pressure for each individual experiment, Pa; $p_{1}$ is the feed side pressure, Pa; P_0_ is the atmospheric pressure, Pa; T is the temperature, K; T_0_ is the 273.15, K; S is the membrane area, m^2^; l is the membrane thickness, m; and τ is the time of experiment, s.

## 3. Results and Discussion

### 3.1. Polymer Characterization

**Figure 3:**
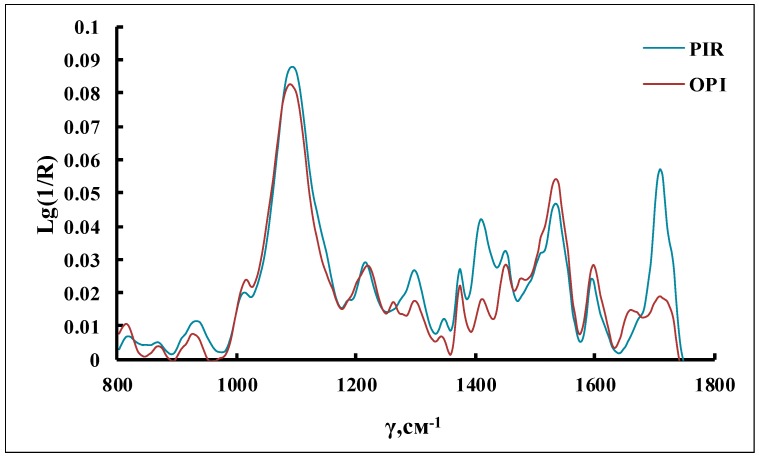
FTIR spectra of the polymers.

It was determined that when the peripheral polyoxyethylene blocks (polyoxypropylene glycol-4000 containing potassium-alcoholate end groups) were not found in MI, the reaction was accompanied by the formation of a glassy polymer product, regardless of the cocatalysts used, and lower temperatures. In that case, polyisocyanurates (PIR) were exclusively formed (Figure 3), according to the FTIR spectra (the presence of analytical bands in the region of 1410 and 1710 cm^−1^).

When MI containing 100% of POE blocks (polyoxyethyleneglycol-4000, containing potassium-alcoholate end groups) is used, the polymer products formed exhibit significant hydrophilicity, low strength characteristics and high plastic deformation. In that case, the formation of O-polyisocyanate structural elements does not occur according to FTIR spectroscopy.

Previous studies [39,40] examined the effect of the molecular weight of block copolymers of propylene and ethylene oxides (the content of peripheral POE blocks was kept constant at 30% for PPEG with molecular weights of 3000, 4200 and 6000 g/mol) on the efficiency of the opening of isocyanate groups as activated on the carbonyl component. They found that the most favorable conditions for the formation of O-polyisocyanate (OPI) structural elements were present when a PPEG with a molecular weight of 4200 g/mol were used. Factors affecting the polyaddition accompanied by the opening of isocyanate groups via the carbonyl component include the use of catalytic amounts of acidic compounds, water in combination with trimethylamine and maintenance of fairly low temperatures [49].

Therefore, the PPEGs with a molecular weight of 4200 g/mol were used to study the effect of the content of peripheral POE blocks on the opening of NCO groups along the C=O link. According to FTIR spectroscopic studies, a change in the content of POE blocks in PPEG from 15 to 30% does not affect the character of the spectra. The bands in the region of 1710 and 1410 cm^−1^ corresponding to the stretching vibrations of the C=O bond are practically absent but a band appeared in the region of 1670 cm^−1^ that corresponds to the stretching vibrations of N=C bonds in O-polyisocyanates (Figure 3). A shoulder observed at 1620 cm^−1^ indicated the formation of urea.

Thus, the presence of terminal POE is a prerequisite for the activation of isocyanate groups under the influence of MI on TDI with their subsequent opening by the carbonyl component. In this regard, the next studies were carried out for polymers obtained on the basis of TDI and PPEG, which contain 15, 20, 30 and 40 wt % of POE blocks.

#### 3.1.1. Thermal and Thermomechanical Behavior

**Figure 4:**
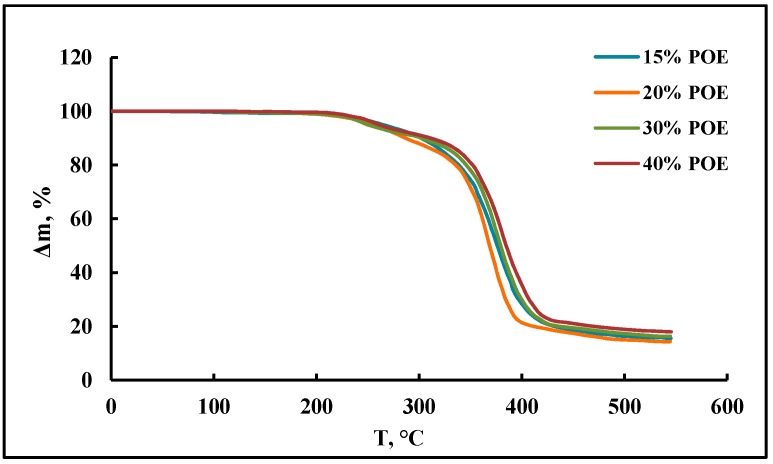
TGA profiles of polymers.

According to the measurements of the thermogravimetric analysis curves (Figure 4), a decrease in the content of POE blocks practically does not affect the thermal stability of polymers.

**Figure 5:**
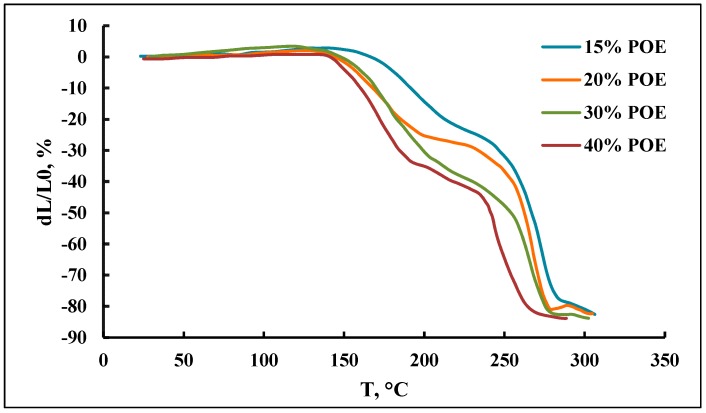
The curves of thermomechanical analysis of polymers.

The curves of thermomechanical analysis (Figure 5) reflect the complexity of the macromolecular and supramolecular organization of the polymers that are being studied. Indeed, the question is about the block copolymers of polyethers and O-polyisocyanates. In turn, the flexible chain (polyether) component represents the block copolymers of propylene and ethylene oxides. Therefore, several relaxation processes are observed on TMA curves, which are significantly influenced by the content of POE blocks in PPEG. With an increase in the weight percentage of POE blocks, a shift in the onset of deformation to a lower temperature region is observed. The observed changes in TMA curves confirm the conclusion that a decrease in the content of POE blocks leads to more rigid packaging of O-polyisocyanate blocks.

In addition, the findings indicate that O-polyisocyanate blocks are the main element of the supramolecular organization of the studied polymers. The domains formed with their participation are distinguished by their stability in high temperatures. Thus, for the polymer obtained at 15 wt % of POE content, the deformation values of 20% are achieved only at 250 °C.

The deformation of the samples, which accompanies the temperature rise from 250 °C, is connected with the processes of thermal destruction of polymers. The run of TMA analysis curves reflects the fact that the segregation of O-polyisocyanate blocks is not due to the intermolecular interactions of physical nature but is instead due to the fact that urea is formed as a result of the involvement in its formation of NCO groups of *ortho*-position TDI, which are included into adjacent O-polyisocyanate blocks.

#### 3.1.2. Mechanical Behavior

**Figure 6:**
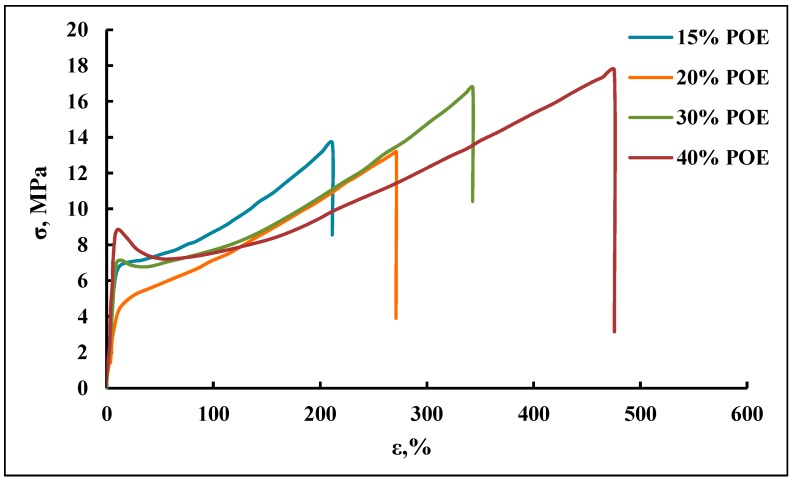
Tensile tests of polymers.

A change in the content of POE blocks leads to noticeable changes in the mechanical behavior of polymers (Figure 6). In this case, there is a change in the run of the curves and a regular increase in the relative elongation at the break of the samples with an increase in the percentage of POE blocks, which reflects the differences in their supramolecular organization. Such changes in the mechanical properties of block copolymers are the consequence of differences in their macromolecular structure. If the POE block content is 15 wt %, the polyoxypropylene segments are located in the internal cavity of voids formed by O-polyisocyanate blocks. When the POE block content is 30 wt %, the flexible chain component forms its own microphase outside the segregation zone of the rigid O-polyisocyanate blocks. As a result, the framework of the supramolecular structure of the resulting block copolymers decreases, leading to an increase in the relative elongation of the film polymer samples at the break.

**Figure 7:**
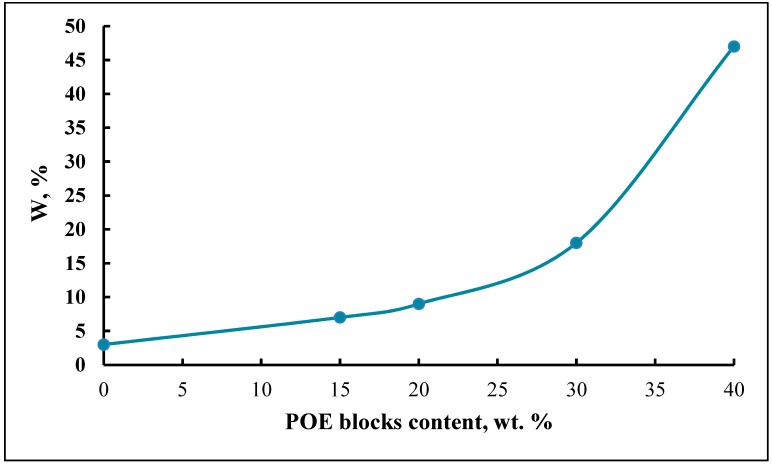
Dependence of the limiting degree of water adsorption (W, %) of polymers on the POE block content (wt %).

It is suggested that during the formation of the cellular supramolecular structure of the synthesized microporous block copolymers, the internal component of the pore depends on the content of POE blocks in the initial macroinitiator. Thus, when the content of POE blocks in PPEG is 15–20 wt %, part of the pore cavity is filled with polyoxypropylene segments. When the content of POE blocks in PPEG is 30 wt %, the surface of the pores is composed of O-polyisocyanate blocks. In these cases, the obtained microporous polymers adsorb water without changing their geometric dimensions. An increase in the content of POE to 40 wt % leads to the swelling of polymer samples in water and the destruction of the cellular supramolecular structure due to the hydrophilicity of the polyoxyethylene segments (Figure 7).

#### 3.1.3. Polymer Surface Morphology

**Figure 8:**
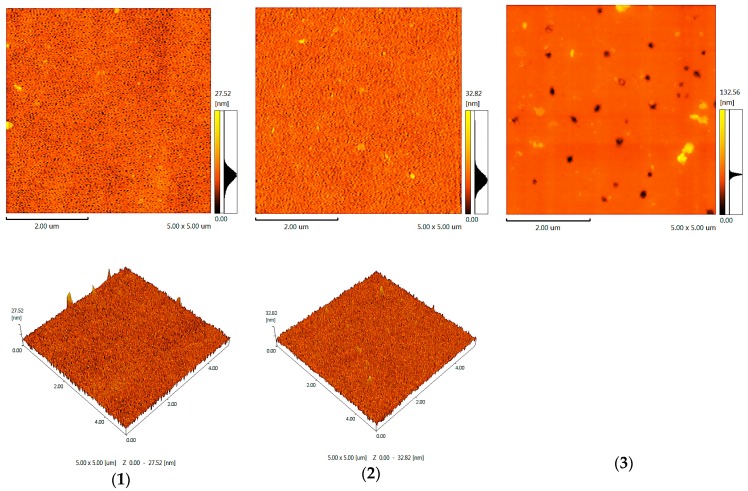
Atomic force microscopy of polymers. The content of POE blocks (wt %) is 15 (**1**); 20 (**2**); and 30 (**3**).

According to the AFM data (Figure 8), the content of POE blocks affects the morphology surface and cavity size. When the content of POE blocks is 20 wt %, the cavity diameter is minimal. When the content of POE blocks is 30 wt %, the cavity diameter increases. The AFM images allowed us to establish that an increase in the POE block content has a disproportionate influence on the nature of changes in the surface morphology of polymers, including the diameter of cavities. Thus, when the POE block content changes from 15 to 20%, the diameter of cavities decreases. When the content of POE blocks is 20 wt %, the cavity diameter is minimal. When the content of the POE is 30 wt %, the cavity diameter significantly increases. In this case, the surface morphology also changes. This is a consequence of the fact that the change in the chemical structure of the flexible chain component in the block copolymer, including coplanar rigid O-polyisocyanate blocks, has a significant impact on the process of their segregation in the macromolecular space.

### 3.2. Gas Transport Properties of Polymers

According to the studies of gas transport characteristics (Table 1, Table 2, Table 3, Table 4, Table 5 and Table 6) of the obtained polymer membrane materials, the permeability for polar molecules of ammonia and hydrogen sulfide significantly exceeds the permeability values obtained for the non-polar molecules of He, N_2_ and CH_4_. A relatively high permeability is also observed for carbon dioxide. The fact that CO_2_ exhibits higher permeability compared to He, N_2_ and CH_4_ is associated with the higher solubility of CO_2_ in polyoxyethylene and polyoxypropylene blocks compared to other gases. This hypothesis is also confirmed by the higher sorption coefficients for CO_2_ in comparison with He, N_2_ and CH_4_ (Table 3). At the same time, the content of POE blocks has a small effect on the permeability for all studied gases. The observed non-monotonous changes in permeability can be explained by the fact that a change in the content of POE blocks leads to significant changes in the packing of the flexible chain component inside of the voids and outside of the segregation of the coplanar O-polyisocyanate blocks. This is why there are not trends formed after increasing the POE content in the obtained polymer. This might also be the reason for the higher diffusion coefficient of the larger molecule of methane compared to the smaller molecule of helium in the case of 20% POE. It is also clear that the solubility coefficient of reactive (acid and base) gases, such as carbon dioxide, hydrogen sulfide and ammonia, is higher than that of helium, nitrogen and methane due to their chemical affinity to the polymeric materials.

It is important to note the high values of ideal selectivity for pairs of gases, including polar and non-polar molecules.

The diffusion coefficient increases with an increase in the POE block content in PPEG for all studied gases. These regularities are consistent with the stated assumption that the voids of polymers obtained with a low content of POE blocks are filled with a polyoxypropylene component. As a result, the space inside the pores is reduced. Therein, for non-polar gases, an obstacle appears due to the imperfection of the channel formation between the pores.

In the case of polar gases, these penetrate as a result of their sorption by chemical substances and subsequently fill the surface of the pores. At the same time, the values of the sorption coefficients for polar gases enormously exceed those for non-polar gases (Table 3).

The established differences in the permeability values turned out to be the cause of the high ideal selectivity for NH_3_/CH_4_, H_2_S/CH_4_, CO_2_/CH_4_, NH_3_/N_2_, H_2_S/He, H_2_S/N_2_, CO_2_/He, NH_3_/He and CO_2_/N_2_ pairs at a POE block content of 15–20 and relatively high ideal selectivity for NH_3_/CO_2_ and H_2_S/CO_2_ pairs. Moreover, the obtained results for CO_2_/N_2_ hold promise in comparison with the data presented on the revised upper bond of Robeson plot [50], especially for the case of 20% POE. For CO_2_/CH_4,_ the best results are obtained with 15% POE but this is slightly lower than the upper bond.

The ability to select the necessary ideal selectivity of the resulting polymer membranes by changing the POE block content in PPEG is important from a practical perspective.

## 4. Conclusions

In this present work, the influence of the content of peripheral POE blocks in PPEG on supramolecular structure processes and gas transport characteristics of polymers obtained on the basis of PPEG and TDI was investigated. The presence of peripheral POE blocks in PPEG is a prerequisite for the activation of isocyanate groups under the influence of MI on TDI, with their subsequent opening by the carbonyl component and formation of O-polyisocyanate blocks.

Several relaxation processes are observed on TMA curves, which are significantly influenced by the content of POE blocks in PPEG. With an increase in the weight percentage of POE blocks, a shift in the onset of deformation to lower temperatures is observed. The observed changes in TMA curves confirm the conclusion that a decrease in the content of POE blocks leads to more rigid packaging of O-polyisocyanate blocks.

From the results obtained in this work, it can be inferred that if the POE block content is 15 wt %, the polyoxypropylene segments are located in the internal cavity of voids formed by O-polyisocyanate blocks. When the POE block content is 30 wt %, the flexible chain component forms its own microphase outside the segregation zone of the rigid O-polyisocyanate blocks.

The permeability for the polar molecules of ammonia and hydrogen sulfide significantly exceeds the permeability values obtained for non-polar molecules of He, N_2_ and CH_4_. A relatively high permeability is also observed for carbon dioxide. At the same time, the content of POE blocks has a small effect on the permeability for all studied gases. The diffusion coefficient increases with an increase in the POE block content in PPEG for all studied gases.

## Figures and Tables

**Table 1:** Polymer permeability coefficient (P) for different single gases obtained at different POE block contents (measurements are given at the pressure of 1 atm).

POE Content, wt %	P, Barrer					
	He	N_2_	CH_4_	CO_2_	NH_3_	H_2_S
15	11	1.5	3	110	587	507
20	5.5	1.7	12	134	563	520
30	19	3.1	11	102	489	454
40	17	2	8	117	693	657

1 Barrer = 3.346 × 10^−16^ mol·m·m^−2^·s^−1^·Pa^−1^.

**Table 2:** Diffusion coefficients (D) of polymer obtained at different POE block contents.

POE Content, wt %	D, 10^−10^, m^2^/s					
	He	N_2_	CH_4_	CO_2_	NH_3_	H_2_S
15	0.10	0.02	0.06	0.28	0.30	0.33
20	0.22	0.22	0.36	0.71	0.62	0.66
30	0.99	0.24	0.53	2.73	2.37	3.10
40	0.84	0.28	0.54	2.89	3.08	4.62

**Table 3:** Sorption coefficients (S) of polymer obtained at different POE block contents.

POE Content, wt %	S, 10^−5^, mol/(m^3^·Pa)					
	He	N_2_	CH_4_	CO_2_	NH_3_	H_2_S
15	35	21	18	133	665	510
20	8.5	3	11	63	303	264
30	65	44	72	125	690	381
40	67	23	48	136	755	476

**Table 4:** Ideal selectivity for NH_3_/gas systems for polymer membranes obtained at different POE block contents.

POE Content, wt %	NH_3_/He			NH_3_/N_2_			NH_3_/CH_4_			NH_3_/CO_2_			NH_3_/H_2_S		
	α_perm_	α_diff_	α_sorp_	α_perm_	α_diff_	α_sorp_	α_perm_	α_diff_	α_sorp_	α_perm_	α_diff_	α_sorp_	α_perm_	α_diff_	α_sorp_
**15**	55	3	19	376	12	32	194	5	38	5	1.06	5	1.16	0.89	1,30
**20**	101	3	36	329	3	115	48	2	28	4	0.88	5	1.08	0.94	1.15
**30**	26	2	11	158	10	16	43	4.5	10	5	0.87	5.5	1.08	0.60	1.81
**40**	41	4	11	352	11	33	89	6	16	6	1.07	5.5	1.06	0.67	1.59

**Table 5:** Ideal selectivity for CO_2_/gas systems for polymer membranes obtained at different POE block contents.

POE Content, wt %	CO_2_/He			CO_2_/N_2_			CO_2_/CH_4_			CO_2_/NH_3_			CO_2_/H_2_S		
	α_perm_	α_diff_	α_sorp_	α_perm_	α_diff_	α_sorp_	α_perm_	α_diff_	α_sorp_	α_perm_	α_diff_	α_sorp_	α_perm_	α_diff_	α_sorp_
**15**	10	2	4	71	11	6	36	5	7.5	0.19	0.94	0.20	0.22	0.84	0.26
**20**	24	3	7	78	3	24	11	2	6	0.24	1.14	0.21	0.26	1.07	0.24
**30**	5	3	2	33	11.5	2	9	5	2	0.21	1.15	0.18	0.22	0.69	0.33
**40**	7	3	2	60	10	6	15	5	3	0.17	0.94	0.18	0.18	0.62	0.29

**Table 6:** Ideal selectivity for H_2_S/gas systems for polymer membranes obtained at different POE block contents.

POE Content, wt %	H_2_S/He			H_2_S/N_2_			H_2_S/CH_4_			H_2_S/CO_2_			H_2_S/NH_3_		
	α_perm_	α_diff_	α_sorp_	α_perm_	α_diff_	α_sorp_	α_perm_	α_diff_	α_sorp_	α_perm_	α_diff_	α_sorp_	α_perm_	α_diff_	α_sorp_
**15**	48	3	14.5	325	13	24	167	5	29	5	1.19	4	0.86	1.13	0.77
**20**	93	3	31	304	3	101	44	2	24	4	0.93	4	0.93	1.06	0.87
**30**	24	4	6	147	17	9	40	8	5	5	1.46	3	0.93	1.68	0.55
**40**	39	5.5	7	334	16	21	84	8.5	10	6	1.60	3.5	0.95	1.5	0.63

## Abbreviations

O-polyisocyanates (OPI)	polyisocyanate of acetal nature
TDI	2,4-toluene diisocyanate
MI	macroinitiator
PPEG	potassium-substituted block copolymers of propylene and ethylene oxide
POE	polyoxyethylene
FTIR	Fourier transform infrared spectroscopy analysis
TMA	thermomechanical analysis
TGA	thermal gravimetric analysis
AFM	atomic force microscopy
PIR	polyisocyanurates
